# Media and Cultural Influences in African-American Girls' Eating Disorder Risk

**DOI:** 10.5402/2013/319701

**Published:** 2013-02-03

**Authors:** Lakaii A. Jones, Catherine Cook-Cottone

**Affiliations:** Department of Counseling, School, and Educational Psychology, Graduate School of Education, University at Buffalo, State University of New York, Buffalo, NY 14620, USA

## Abstract

*Objective*. To investigate media and cultural influences in eating disorder development in African-American adolescent females. *Method*. Fifty-seven participants were recruited through churches and community organizations to complete a questionnaire. *Results*. Mainstream sociocultural identification was associated with more eating disorder behavior in African-American females; cultural ethnic identification was not significantly associated with eating disorder behavior in African-American females, mainstream sociocultural identification, cultural ethnic identification, and body dissatisfaction significantly predicted eating disorder behavior; and cultural ethnic identification was positively correlated with mainstream sociocultural identification. This study provides support for the importance of eating disorder prevention interventions that focus specifically on African-American girls.

## 1. Introduction

The research on eating disorders (EDs) in minorities, specifically African-Americans, is scarce. Even rarer is literature that examines the cultural and media influences of eating pathology in this population, specifically in those from urban areas. There is also little research on African-American middle school and early high school age females. Few studies focus on middle school adolescents [1, 2]. Many of the studies on general EDs and media influences on EDs focus on college students [3, 4]. Some findings suggest that within minority populations, eating disorder (ED) development is considered rare (e.g., [5]). However, recently such findings have come into question [6] and few studies have used solely minority populations in investigating ED risk [4, 5].

The Diagnostic and Statistical Manual of Mental Disorders Fourth Edition-Text Revision (DSM-IV-TR) describes three types of EDs, anorexia nervosa (AN), bulimia nervosa (BN), and Eating Disorder-Not Otherwise Specified (ED-NOS). In AN, individuals refuse to sustain normal body weight;, in BN, individuals binge eat and compensate for binging using inappropriate methods (e.g., self-induced vomiting, excessive exercise), in order to maintain weight, and in ED-NOS, individuals do not meet the complete criteria for an ED but display features of either AN or BN [7]. AN and BN are found to be less common in African-American youth (In their review of the literature, Anthony and Yager [8] stated that various studies), [9]. However, the aforementioned studies did not specifically focus on middle school and early high school age African-American females living in an urban area. 

Compared with Caucasians, Binge Eating Disorder (BED) is shown to be the most common ED among African American adolescents and adults [10]. BED is the consumption of large quantities of food in a short period of time accompanied by a feeling of lack of control over eating behavior [11]. Thus, investigating BED behavior in addition to AN and BN behaviors is important. 

Notably, a large part of ED development in America is exposure to the media [2, 12]. Particularly, African-American females watch more television than White females [13]. American culture uses the media to promote a thin ideal, which may be a risk factor in the development of EDs, specifically for minorities [2]. Much of the research examining African-American female youth has compared them to Caucasians [13]. Thus, more research needs to be done on media impact specifically in African-American girls living in an urban area.

Nevertheless, despite the dearth of research about this specific population, the field is making progress in targeting minority females living in urban areas [14]. There have been many gains in the field of EDs in examining cultural and media influences, as well as risk and protective factors in ED symptom and disease development [15]. However, there is still much room for improvement in examining African-American females living in urban areas and investigating the correlates of media and cultural influences. Thus, due to gaps in the aforementioned literature, there was a need to conduct research that utilized an all minority, female, middle school to early high school age sample living in an urban area. For the purposes of this study, African-American girls were the focus. 

## 2. Cultural Manifestations of Eating Disorders

In assessing cultural aspects of EDs in minority populations, a review of research has shown that compared to Caucasian females, eating pathology is more common among Native American females, equally common among Hispanic females, and less common in Asian American and Black adolescents [16]. However, those minorities with a high acculturation to Western culture “the process of shifting values to the host culture from the culture of origin” [12, page 19] are possibly more at risk for developing EDs because they have internalized the thin ideal notion of beauty [12]. 

In a study of seven African-American and Latina adolescent females, high acculturation to Western culture contributed to eating disorder development [5]. Other research examined internalization of American values in college women [3, 17]. Internalization of American values was found to be related to identification with White culture and less satisfaction with body shape and size. A combination of DT, physical self-concept, and personal competence greatly predicts body dissatisfaction (BD) [3, 17]. This finding is consistent with a study of young black, Hispanic, and White women and men. Demarest and Allen [18] found that African American females (mean dissatisfaction score = 9.4, SD = 14.4) desired thinner bodies and were not more satisfied with their bodies than Caucasian females (mean dissatisfaction score = 13.5, SD = 13.1). However, none of these aforementioned studies examined African-American middle school to early high school age students living in an urban setting. 

## 3. Media Influences on Eating Disorders

Very few studies have specifically examined African-American female adolescents and media influences, highlighting the need for more research in this population. One example is the aforementioned Botta [13] that examined television watching in 33 Black and 145 White female adolescents, finding that Black girls participated in more disordered eating behaviors than White girls. The more adolescents viewed television shows loaded with thin images, the more likely they participated in disordered eating behaviors, the more dissatisfied they were with their bodies, and the greater their drive for thinness (DT) [13]. The closest to a causal link between media exposure and disordered eating behavior is studies done in Fiji on ethnic Fijian girls. These studies examined the impact introduction of television in the country (where television had not previously been) on adolescent Fijian high school girls' body image perception and development of eating disordered behaviors [6, 19]. Results revealed a significant increase in the salience of self-induced purging behavior for weight loss and high Eating Attitudes Test (EAT) scores following extended television exposure in Fiji in 63 girls in 1995 and 65 girls in 1998 [6, 19]. 

## 4. Risk Factors in Eating Disorders

### 4.1. Body Dissatisfaction

In minority females, evidence for the influence of thin ideal internalization on BD has been found [20, 21]. DT predicts BD in minorities [3, 17, 18]. Furthermore, BD has been found to be the single strongest predictor of ED symptomatology [22]. Higher acculturation to Western values can cause minority adolescent females to internalize thin media figures [12]. 

### 4.2. Drive for Thinness

 In both White and Black adolescent girls, fat has been found to be related to DT. In African American girls, it has been a predictor of criticisms about weight [23]. For young women without AN or BN, greater emphasis on shape and weight has been found to predict DT in females [24]. Thus, investigating DT in African-American girls is important in understanding how it affects eating behavior.

## 5. Summary and Hypotheses

This study aimed to examine media and cultural influences in ED development in African-American, female, middle school to early high school age girls living in an urban area. It was hypothesized that (a) mainstream sociocultural identification will be associated with more ED behavior in African-American females; (b) cultural ethnic identification will be associated with less ED behavior in African-American females; (c) mainstream sociocultural identification, cultural ethnic identification, and BD will significantly predict ED behavior in African-American females; and (d) cultural ethnic identification will be negatively correlated with mainstream sociocultural identification in African-American females. The influence of body mass index (BMI) was also examined in relation to ED development in this study, although no specific hypothesis was proposed regarding this influence.

## 6. Method

### 6.1. Participants

Participants (*N* = 57) were recruited from community organizations, churches, and neighborhoods in an urban Midwest City and an urban east coast city. Of these 57 participants, 50 (*n* = 50) met criteria for participation in the study. All of the qualified participants self-identified as African-American. Of the seven girls did not meet criteria for this study, four self-identified as Hispanic, one self-identified as Native American, one was African-American but did not have proper parent consent, and one was a nine-year-old African-American girl who also did not have proper parent consent.

The girls ranged in age from 10 to 15 years old (*M* = 12.54, SD = 1.57). They were in grades from 4 to 10 (*M* = 7.35, SD = 1.59). They ranged from a height of 55 inches to 71 inches (*M* = 62.07, SD = 3.41). Their weight ranged from 53 to 160 pounds (*M* = 117.69, SD = 26.76). BMI was calculated on 38 individuals (76%) based on self-reported height and weight and ranged from 14.40 to 31.30 (*M* = 21.88, SD = 4.01). Father's education and mother's education were coded as follows as a measure of socioeconomic status (SES): 1 = less than high school, 2 = high school graduate, 3 = one to three years of college, 4 = college graduate, and 5 = graduate school. Father's education was calculated on 38 individuals (76%) and ranged from 1 to 5 (*M* = 2.95, SD = 1.14). Mother's education also ranged from 1 to 5 (*M* = 3.25, SD = 1.19) and was calculated on 40 participants (80%).

### 6.2. Measures

#### 6.2.1. Demographic Questionnaire

This questionnaire asked participants' current age, grade, height, weight, race/ethnicity, and parents'/guardians' highest level of education. 

#### 6.2.2. Eating Disorder Inventory-3

 The Eating Disorder Inventory-3 (EDI-3) [25] is a measure utilized to detect individuals at risk for EDs. Three subscales of the EDI-3 were used: DT, Bulimia, and BD. DT measures excessive worries about dieting, fixation with weight, and involvement in an intense quest of thinness. Bulimia measures the tendency toward binge eating followed by self-induced vomiting and other compensation methods. BD measures the belief that particular body parts (e.g., thighs, stomach, and hips) are too big [25]. Reliability was established on a sample of American adolescents, international adults, and American adults. Reliability for the DT and BD scales ranged from 0.90 to 0.97 across the three groups. Alpha Coefficients of the other subscales had medians of 0.74, 0.84, and 0.85. Internal consistency was found to be good. Construct validity at its highest was 0.83. The DT and Bulimia scales ask questions such as “I stuff myself with food” and “If I gain a pound, I worry that I will keep gaining” [26]. Although the EDI-3 was designed for females ages 13–53, a previous version of the EDI has been successfully used with younger adolescents [22], and the scales are written at a fourth to sixth grade reading level [26]. For this study, Cronbach's alpha is 0.77 for the DT scale, 0.72 for the Bulimia scale, and 0.83 for the BD scale.

#### 6.2.3. Adolescent Version of the Questionnaire of Eating and Weight Patterns

 The adolescent version of the Questionnaire of Eating and Weight Patterns (QEWP-A) [11] is a 12-question self-report measure designed to assess BED in individuals. Psychometric properties of the QEWP-A were assessed on a sample of 6th to 12th graders ranging in age from 10 to 18 years. Construct validity was adequate when compared with other measures of eating behaviors [27]. Questions asked include, “During the past 6 months, did you ever eat what most people, like your friends, would think was a *really big* amount of food?” [27, page 306] and “Did you ever *not eat anything at all* for at least *24 hours* (a full day) to keep from gaining weight after eating a *really big *amount of food?” [27, page 308]. For the purposes of this study, this scale was split into two separate scales, binge behaviors and compensatory behaviors. This was done in order to distinguish binge eating behaviors from compensatory methods. For this study, the Cronbach's alpha for the QEWP-A is 0.70.

#### 6.2.4. Sociocultural Attitudes Towards Appearance Questionnaire-3

The Sociocultural Attitudes Towards Appearance Questionnaire-3 (SATAQ-3) [28], which looks at media and social influences on appearance and body image, was originally normed with college women (found was good convergent validity, sufficient internal consistency, and replicable factor structure) but was assessed on middle school boys and girls [29]. The SATAQ-3 is a 30-question scale with two subscales, Awareness and Internalization of a thin body ideal. Girls in the study were given a modified version of the SATAQ-3. These subscales displayed sufficient internal consistency, adequate validity, and strong correlations with body image and the use of weight control methods, particularly the Internalization subscale. However, the scale assessed a nearly all White samples [29]. Nevertheless, for the current study, reliability was examined for African-American middle school to early high school age girls. For this study, the Cronbach's alpha for the SATAQ-3 is 0.89.

#### 6.2.5. Multigroup Ethnic Identity Measure

 The Multigroup Ethnic Identity Measure (MEIM) [30], a 14-item questionnaire examining ethnic affirmation and belonging ethnic identity achievement, ethnic behaviors, and 6 additional questions on group orientation, was developed to measure ethnic identity in African American, Native American, Caucasian, Asian-American, and Hispanic adolescents and adults [30, 31]. Cronbach's alpha found reliability to be 0.81 for the adolescents and 0.90 for college students assessed [30]. Two subscales of the MEIM have been assessed in African American middle school girls (ages from 11 to 14 years), Ethnic Identity Exploration and Ethnic Identity Commitment. Found was good reliability and internal consistency with the Ethnic Identity Commitment subscale. The subscale was also found to be replicable [32]. For this study, the Cronbach's alpha for the MEIM is 0.80.

### 6.3. Statistical Analysis

Data was analyzed via simple linear regression, which determined a relationship between the independent and dependent variables of each study hypothesis. Hierarchical linear regression was also used examining stepwise regression. Linear regression was used because often this method can be used to predict the value of one variable from the value of another [33]. Specifically, Pearson Product Moment Correlation Coefficient was used due to its ability to denote both the strength and the direction of the relationship between two variables [34]. A correlation matrix was constructed for the data (see Table 1).

## 7. Results

### 7.1. Hypothesis One

The correlation between mainstream sociocultural identification and DT was positive and significant (*r* = 0.449, *P* = 0.001). As the mainstream sociocultural identification increases, DT increases, and there is a significant relationship between the two, *β* = 0.449; *t* = 3.485; *P* = 0.001. Mainstream sociocultural identification explained a significant proportion of variance in DT, *R* ^2^ = 0.202; *F*(1, 48) = 12.148; *P* = 0.001. The correlation between mainstream sociocultural identification and BD was positive and significant (*r* = 0.365, *P* = 0.009). As the mainstream sociocultural identification increases, BD increases, and there is a significant relationship between the two, *β* = 0.365; *t* = 2.713; *P* = 0.009. Mainstream sociocultural identification explained a significant proportion of variance in BD, *R* ^2^ = 0.133; *F*(1, 48) = 7.363; *P* = 0.009. The correlation between mainstream sociocultural identification and BED symptomatology was positive and significant (*r* = 0.281, *P* = 0.048). As mainstream sociocultural identification increases, BED symptomatology increases, and there is a significant relationship between the two, *β* = 0.281; *t* = 2.031; *P* = 0.048. Mainstream sociocultural identification explained a significant proportion of variance in BED symptomatology, *R* ^2^ = 0.079; *F*(1, 48) = 4.125; *P* = 0.048. In examining the splitting of the QEWP-A questionnaire into two questionnaires, binge behavior and compensatory behaviors, the correlation between mainstream sociocultural identification and compensatory behaviors was positive and significant (*r* = 0.547, *P* < 0.001). As mainstream sociocultural identification increases, compensatory behaviors increases, and there is a significant relationship between the two, *β* = 0.547; *t* = 4.530; *P* < 0.001. Mainstream sociocultural identification explained a significant proportion of variance in compensatory behaviors, *R* ^2^ = 0.299; *F*(1, 48) = 20.522; *P* < 0.001. However, there was neither significant correlation between mainstream sociocultural identification and binge eating behaviors nor a significant correlation between mainstream sociocultural identification and Bulimia (see Table 2).

 A hierarchical linear regression was also done of hypothesis one. Two steps were entered. The first step was BMI because the purpose was to control for this variable. The second step was mainstream sociocultural identification. BMI was a significant predictor of DT (*F*(1, 36) = 4.277, *P* = 0.046). BMI explained a significant proportion of variance in DT, Δ*R* ^2^ = 0.106. Mainstream sociocultural identification was also a significant predictor of DT (*F*(1, 35) = 17.501, *P* < 0.001) and there is a significant relationship between the two, *β* = 0.572; *t* = 4.183; *P* < 0.001, even after controlling for the effect of BMI. Mainstream sociocultural identification explained a significant proportion of variance in DT, Δ*R* ^2^ = 0.298, *P* < 0.001. BMI was a significant predictor of BD (*F*(1, 36) = 5.792, *P* = 0.021). BMI explained a significant proportion of variance in BD, Δ*R* ^2^ = 0.139; *P* = 0.021. Mainstream sociocultural identification was also a significant predictor of BD (*F*(1, 35) = 7.973, *P* = 0.008), and there is a significant relationship between the two, *β* = 0.419; *t* = 2.824; *P* = 0.008, above and beyond the effect of BMI. Mainstream sociocultural identification explained a significant proportion of variance in BD, Δ*R* ^2^ = 0.160, *P* = 0.008.

BMI was not a significant predictor of BED symptomatology. However, mainstream sociocultural identification was a significant predictor of BED symptomatology (*F*(1, 35) = 6.966, *P* = 0.012), and there is a significant relationship between the two, *β* = 0.426; *t* = 2.639; *P* = 0.012, even after controlling for the effect of BMI. Mainstream sociocultural identification explained a significant proportion of variance in BD, Δ*R* ^2^ = 0.165, *P* = 0.012. BMI was not a significant predictor of compensatory behaviors. However mainstream sociocultural identification was a significant predictor of compensatory behaviors (*F*(1, 35) = 12.450, *P* = 0.001), and there is a significant relationship between the two, *β* = 0.527; *t* = 3.528; *P* = 0.001, above and beyond the effect of BMI. Mainstream sociocultural identification explained a significant proportion of variance in compensatory behaviors, Δ*R* ^2^ = 0.253, *P* = 0.001. Neither BMI, nor mainstream sociocultural identification was a significant predictor of Bulimia or binge behaviors.

### 7.2. Hypothesis Two

There was no significant correlation between cultural ethnic identification and DT (*r* = 0.114, *P* = 0.429). There was no significant correlation between cultural ethnic identification and Bulimia (*r* = 0.081, *P* = 0.576). There was no significant correlation between cultural ethnic identification and BD (*r* = 0.149, *P* = 0.300). There was no significant correlation between cultural ethnic identification and BED symptomatology (*r* = 0.014, *P* = 0.923). There was no significant correlation between cultural ethnic identification and binge behaviors (*r* = 0.109, *P* = 0.452). There was no significant correlation between cultural ethnic identification and compensatory behaviors (*r* = 0.083, *P* = 0.567). A hierarchical linear regression was also done for hypothesis two. Two steps were entered. The first step was BMI because the purpose was to control for this variable. The second step was cultural ethnic identification. Cultural ethnic identification was not a significant predictor of DT (*F*(1, 35) = 2.811, *P* = 0.074), Bulimia (*F*(1, 35) = 1.081, *P* = 0.900), BD (*F*(1, 35) = 3.301, *P* = 0.367), BED symptomatology (*F*(1, 35) = 0.086, *P* = 0.918), binge behaviors (*F*(1, 35) = 483, *P* = 0.621), or compensatory behaviors (*F*(1, 35) = 0.724, *P* = 0.492), even after controlling for BMI. 

### 7.3. Hypothesis Three

A hierarchical linear regression was done for hypothesis three. Four steps were entered. The first step was BMI because the purpose was to control for this variable. The second step was cultural ethnic identification because it was deemed the least important variable in ED symptomatology. The third step was mainstream sociocultural identification because it was postulated to be the second most important factor in ED behavior. The last step was BD because it was deemed the most important variable in ED symptomatology. 

Results revealed that mainstream sociocultural identification was a significant predictor of DT (*F*(1, 34) = 15.918, *P* = 0.000), and there is a significant relationship between the two, *β* = 0.371; *t* = 2.056; *P* = 0.048, even after controlling for BMI and cultural ethnic identification. Mainstream sociocultural identification explained a significant proportion of variance in DT, Δ*R* ^2^ = 0.275, *P* = 0.000. Results also revealed that BD was still a significant predictor of DT (*F*(1, 33) = 5.897, *P* = 0.021), and there is a significant relationship between the two, *β* = 0.400; *t* = 2.428; *P* = 0.021, above and beyond the effects of BMI, cultural ethnic identification, and mainstream sociocultural identification. BD explained a significant proportion of variance in DT, Δ*R* ^2^ = 0.089, *P* = 0.021.

Mainstream sociocultural identification was a significant predictor of BED symptomatology (*F*(1, 34) = 9.495, *P* = 0.004). Mainstream sociocultural identification explained a significant proportion of variance in BED symptomatology, Δ*R* ^2^ = 0.217, *P* = 0.004. However, mainstream sociocultural identification was not a significant predictor of BED symptomatology after controlling for BMI and cultural ethnic identification. BD was also not a significant predictor of BED symptomatology after controlling for BMI, cultural ethnic identification, and mainstream sociocultural identification. Mainstream sociocultural identification was a significant predictor of compensatory behaviors (*F*(1, 34) = 15.092, *P* < 0.001), and there is a significant relationship between the two, *β* = 0.524; *t* = 2.553; *P* = 0.015, above and beyond the effects for BMI and cultural ethnic identification. Mainstream sociocultural identification explained a significant proportion of variance in compensatory behaviors, Δ*R* ^2^ = 0.295, *P* ≤ 0.001.

Mainstream sociocultural identification was not a significant predictor of Bulimia even after controlling for BMI and cultural ethnic identification. BD was also not a significant predictor of Bulimia even after controlling for BMI, cultural ethnic identification, and mainstream sociocultural identification. BD did not predict BED symptomatology after controlling for BMI, cultural ethnic identification, and mainstream sociocultural identification. Mainstream sociocultural identification was not a significant predictor of binge behavior even after controlling for BMI and cultural ethnic identification. BD was also not a significant predictor of binge behavior even after controlling for BMI, cultural ethnic identification, and mainstream sociocultural identification. Lastly, BD did not predict compensatory behaviors above and beyond BMI, cultural ethnic identification, and mainstream sociocultural identification.

### 7.4. Hypothesis Four

The correlation between cultural ethnic identification and mainstream sociocultural identification was positive and significant (*r* = 0.432, *P* = 0.002). As cultural ethnic identification increases, mainstream sociocultural identification increases, and there is a significant relationship between the two, *β* = 0.432; *t* = 3.316; *P* = 0.002. Cultural ethnic identification explained a significant proportion of variance in mainstream sociocultural identification, *R* ^2^ = 0.186; *F*(1, 48) = 10.994; *P* = 0.002 (see Table 3).

## 8. Discussion

To date, to the researcher's knowledge, no research study has examined media and cultural influences in African-American middle school to early high school age girls living in an urban setting. Thus, this study has contributed to the existing, albeit limited, literature on African-American females by showing the effects of acculturation on African-American females residing in urban settings. Similar in this study to other studies is the sample size being comparable to other studies that utilize Black girls, particularly in gathering approximately 50 participants [35, 36]. The SES of participants and the utilization of middle school to high school age adolescents are also comparable to the current study [36–38]. The results from the current study suggest that in African-American girls living in an urban, primarily lower-middle class setting, the perceptions of American beauty standards and thin ideal may arise similarly as in upper-middle class, suburban minority populations [5, 12]. 

Specifically, this study found that as mainstream sociocultural identification increases, DT increases (*r* = 0.449, *P* = 0.001); as mainstream sociocultural identification increases, body dissatisfaction increases (*r* = 0.365, *P* = 0.009); as mainstream sociocultural identification increases, BED symptomatology increases (*r* = 0.281, *P* = 0.048); as mainstream sociocultural identification increases, compensatory behaviors increases (*r* = 0.547, *P* < 0.001). These findings are consistent with other research that suggests that thin ideal media exposure in adolescents is associated with body dissatisfaction and eating disorder symptomatology [19, 39, 40]. 

Nevertheless, in this study there was neither significant correlation between mainstream sociocultural identification and solely binge eating behaviors nor any significance between mainstream sociocultural identification and Bulimia. The former finding contradicts findings that heavy television viewing (i.e., images encouraging viewers to consume and purchase unhealthy foods) in children is related to binge eating behavior [41], while the latter finding contradicts findings that mainstream sociocultural identification is related to Bulimia symptoms in young girls [42]. Perhaps the preponderance of thin ideal images in the media are more influential in compensatory and dieting behaviors for this population, consistent with the strong effect of these images in other research [41]. Furthermore, in Bulimia, other research has shown that exposure to overweight television and magazine figures is correlated with Bulimia [40]. Thus, exposure to thin images may have overridden the effect of exposure to overweight images in this sample [12].

No support was found in cultural ethnic identification being associated with less ED behavior in African-American females, contradicting research that suggests that minority girls with less identification with Western culture have lower risk of ED development [5, 12]. Also contrary to predictions, cultural ethnic identification was positively correlated with mainstream sociocultural identification. This finding is especially puzzling in light of research and theory that suggests that cultural ethnic identification is a protective factor in rejecting Western thin ideal [5, 43]. However, in this sample, none of these girls were from outside of Western culture. Thus, they likely endorsed the values of American culture. Individuals who have a high acculturation to American culture are possibly more at risk for developing ED symptomatology because they have internalized the thin ideal notion of beauty [12]. 

## 9. Limitations

There are several limitations of this study that should be noted. The first limitation is that this sample was a volunteer sample recruited through predominately African-American groups. This recruitment method might have affected the results in the effect of cultural ethnic identification in this sample. For example, the range of scores (*M* = 36.21, SD = 5.08) may have been restricted due to the degree of cultural identification manifest in girls strongly associated with a cultural group. Without a wide range in responses, the results may have been attenuated. Nevertheless, other studies examining this hard-to-reach population have recruited a nonrandom sample of urban youth from community organizations serving these specific youth and Black neighborhoods [44, 45].

A second limitation is that a self-report measure was used, which could have affected accuracy of demographic information (e.g., height, weight, and parents' education). This method may also have lent itself to response bias. A third limitation is that BMI was not collected on all of the girls (24%), which could have influenced the results BMI had on ED symptomatology in this study. A fourth limitation of this study is that even girls who identified as African-American and one or more other racial group were counted as African-American for the purposes of this study. Biracial or multiethnic identity could have impacted findings in some unknown way (20% of girls identified as Biracial or multiethnic). A fifth limitation of this study is that the age range of the girls varied. This sample represented the range of adolescence. Future studies should look at stages of adolescence as a possible factor. Finally, this sample was derived from a generally urban setting. As a result, findings may not be generalizable to African-American girls in other demographics.

## 10. Implications

This study has implications for working with African-American girls in preventing ED behavior, specifically in school-based groups. Many ED prevention mechanisms for adolescent girls are school-based groups which can reach a large number of girls [46], and several have found some effectiveness in preventing ED behavior in adolescent girls [38]. However, most of these groups do not focus specifically on African-American adolescent girls [38, 47]. Nevertheless, one study suggests that minority and nonminority middle school girls do not differ in response to an ED prevention group [36]. 

## 11. Conclusion

Overall, further research is needed in this area in investigating ED prevention in African-American middle school to early high school age girls living in an urban setting. The current study shows the importance of mainstream sociocultural identification in the development of ED symptomatology and how cultural ethnic identification is not as important when girls are already part of a set cultural ethnic group. Essentially, this study is a small step in alerting researchers to the need for more studies in this area.

## Figures and Tables

**Table 1:** Correlation matrix.

Measure	1	2	3	4	5	6	7	8	9
(1) RAWBMI	—	0.33*	0.24	0.37*	0.12	0.30	0.07	0.14	0.19
(2) DT		—	0.13	0.59**	0.11	0.45**	0.19*	−0.09	0.38**
(3) BU			—	0.17	−0.08	0.21	0.57**	0.38**	0.49**
(4) BD				—	−0.15	0.37**	0.35*	0.11	0.42**
(5) MEIM					—	0.43**	−0.01	−0.11	0.08
(6) SATAQ-3						—	0.28*	−0.13	0.55**
(7) QEWP-A							—	0.76**	0.78**
(8) BINGE								—	0.18
(9) COMPENSATE									—

*P < 0.5.  **P < 0.01.

DT: drive for thinness.

BU: Bulimia.

BD: body dissatisfaction.

MEIM: Multigroup Ethnic Identity Measure.

SATAQ-3: Sociocultural Attitudes Towards Appearance Questionnaire-3.

QEWP-A: Questionnaire of Eating and Weight Patterns-Adolescent.

**Table 2:** Hypothesis One: simple linear regression with mainstream sociocultural identification as a predictor variable.

Variable	*β*	*R* ^2^	*F *	Δ*R* ^2^	*t *	Sig. (*P*)	95% CI
DT	0.45	0.20	12.15	0.19	3.49	0.001**	[0.20, 0.65]
BD	0.37	0.13	7.36	0.12	2.71	0.009**	[0.10, 0.58]
BED symptomatology	0.28	0.08	4.13	0.06	2.03	0.048*	[0.00, 0.52]
COMPENSATE	0.55	0.30	20.52	0.28	4.53	0.000**	[0.32, 0.72]

*P < 0.05. **P < 0.01.

DT: drive for thinness.

BD: body dissatisfaction.

BED symptomatology: Binge Eating Disorder.

CI: Confidence Interval.

**Table 3:** Hypothesis Four: simple linear regression with cultural ethnic identification as a predictor variable.

Variable	*β*	*R* ^2^	*F *	Δ*R* ^2^	*t *	Sig. (*P*)	95% CI
Media	0.43	0.19	10.99	0.17	3.32	0.002**	[0.18, 0.63]

**P < 0.01.

CI: Confidence Interval.

## References

[1] Lawrie Z, Sullivan EA, Davies PSW, Hill RJ (2006). Media influence on the body image of children and adolescents. *Eating Disorders*.

[2] Utter J, Neumark-Sztainer D, Wall M, Story M (2003). Reading magazine articles about dieting and associated weight control behaviors among adolescents. *Journal of Adolescent Health*.

[3] Lester R, Petrie TA (1998). Physical, psychological, and societal correlates of bulimic symptomatology among African American college women. *Journal of Counseling Psychology*.

[4] Mulhollanda AM, Mintz LB (2001). Prevalence of eating disorders among African American women. *Journal of Counseling Psychology*.

[5] Silber TJ (1986). Anorexia nervosa in blacks and hispanics. *International Journal of Eating Disorders*.

[6] Becker AE (2004). Television, disordered eating, and young women in Fiji: negotiating body image and identity during rapid social change. *Culture, Medicine and Psychiatry*.

[7] American Psychiatric Association (2000). *Diagnostic and Statistical Manual of Mental Disorders*.

[8] Anthony TM, Yager J, Yager J, Powers PS (2007). Cultural considerations in eating disorders. *Clinical Manual of Eating Disorders*.

[9] Crago M, Shisslak CM (2003). Ethnic differences in dieting, binge eating, and purging behaviors among American females: a review. *Eating Disorders*.

[10] Taylor JY, Caldwell CH, Baser RE, Faison N, Jackson JS (2007). Prevalence of eating disorders among blacks in the National Survey of American Life. *International Journal of Eating Disorders*.

[11] Spitzer RL, Devlin M, Walsh BT (1992). Binge eating disorder: a multisite field trial of the diagnostic criteria. *International Journal of Eating Disorders*.

[12] Kempa ML, Thomas AJ (2000). Culturally sensitive assessment and treatment of eating disorders. *Eating Disorders*.

[13] Botta RA (2000). The mirror of television: a comparison of Black and White adolescents’ body image. *Journal of Communication*.

[14] Gentile K, Raghavan C, Rajah V, Gates K (2007). It doesn’t happen here: eating disorders in an ethnically Diverse sample of economically disadvantaged, urban college students. *Eating Disorders*.

[15] Arnett JJ (2007). *Encyclopedia of Children, Adolescents, and the Media*.

[16] Crago M, Shisslak CM, Estes LS (1996). Eating disturbances among American minority groups: a review. *International Journal of Eating Disorders*.

[17] James KA, Phelps L, Bross AL (2001). Body dissatisfaction, drive for thinness, and self-esteem in african american college females. *Psychology in the Schools*.

[18] Demarest J, Allen R (2000). Body image: Gender, ethnic and age differences. *Journal of Social Psychology*.

[19] Becker AE, Burwell RA, Gilman SE, Herzog DB, Hamburg P (2002). Eating behaviours and attitudes following prolonged exposure to television among ethnic Fijian adolescent girls. *British Journal of Psychiatry*.

[20] Austin JL, Smith JE (2008). Thin ideal internalization in mexican girls: a test of the sociocultural model of eating disorders. *International Journal of Eating Disorders*.

[21] Warren CS, Gleaves DH, Cepeda-Benito A, Fernandez MDC, Rodriguez-Ruiz S (2005). Ethnicity as a protective factor against internalization of a thin ideal and body dissatisfaction. *International Journal of Eating Disorders*.

[22] Phelps L, Johnston LS, Augustyniak K (1999). Prevention of eating disorders: identification of predictor variables. *Eating Disorders*.

[23] Striegel-Moore RH, Schreiber GB, Pike KM, Wilfley DE, Rodin J (1995). Drive for thinness in black and white preadolescent girls. *International Journal of Eating Disorders*.

[24] Anderson CB, Bulik CM (2004). Gender differences in compensatory behaviors, weight and shape salience, and drive for thinness. *Eating Behaviors*.

[25] Garner DM, Olmstead MP, Polivy J (1982). Development and validation of a multidimensional eating disorder inventory for anorexia nervosa and bulimia. *International Journal of Eating Disorders*.

[26] Atlas JA, Spies RA, Plake BS, Geisinger KF, Carlson JF Review of the eating disorder inventory-3.

[27] Johnson WG, Grieve FG, Adams CD, Sandy J (1999). Measuring binge eating in adolescents: adolescent and parent versions of the questionnaire of eating and weight patterns. *International Journal of Eating Disorders*.

[28] Heinberg LJ, Thompson JK, Stormer S (1995). Development and validation of the sociocultural attitudes towards appearance questionnaire. *International Journal of Eating Disorders*.

[29] Smolak L, Levine MP, Thompson JK (2001). The use of the sociocultural attitudes towards appearance questionnaire with middle school boys and girls. *International Journal of Eating Disorders*.

[30] Phinney J (1992). The multigroup ethnic identity measure: a new scale for use with diverse groups. *Journal of Adolescent Research*.

[31] Worrell FC (2000). A validity study of scores on the Multigroup Ethnic Identity Measure based on a sample of academically talented adolescents. *Educational and Psychological Measurement*.

[32] Pegg PO, Plybon LE (2005). Toward the theoretical measurement of ethnic identity. *Journal of Early Adolescence*.

[33] Hurlburt RT (2006). *Comprehending Behavioral Statistics*.

[34] Ary D, Jacobs LC, Razavieh A, Sorensen C (2006). *Introduction to Research in Education*.

[35] Caradas AA, Lambert EV, Charlton KE (2001). An ethnic comparison of eating attitudes and associated body image concerns in adolescent South African schoolgirls. *Journal of Human Nutrition and Dietetics*.

[36] Cook-Cottone C, Jones LA, Haugli S (2010). Prevention of eating disorders among minority youth: a matched-sample repeated measures study. *Eating Disorders*.

[37] Barry DT, Grilo CM (2002). Eating and body image disturbances in adolescent psychiatric inpatients: gender and ethnicity patterns. *International Journal of Eating Disorders*.

[38] Scime M, Cook-Cottone C (2008). Primary prevention of eating disorders: a constructivist integration of mind and body strategies. *International Journal of Eating Disorders*.

[39] Hargraves DA, Tiggemann M (2003). Longer-term implications of responsiveness to ’thin-ideal’ television: support for a cumulative hypothesis of body image disturbance?. *European Eating Disorders Review*.

[40] Harrison K (2000). The body electric: thin-ideal media and eating disorders in adolescents. *Journal of Communication*.

[41] Jordan A (2004). The role of media in children’s development: an ecological perspective. *Journal of Developmental and Behavioral Pediatrics*.

[42] Shorter L, Brown SL, Quinton SJ, Hinton L (2008). Relationships between body-shape discrepancies with favored celebrities and disordered eating in young women. *Journal of Applied Social Psychology*.

[43] French SE, Seidman E, Allen L, Aber JL (2006). The development of ethnic identity during adolescence. *Developmental Psychology*.

[44] Barnett D, Kidwell SL, Leung KH (1998). Parenting and preschooler attachment among low-income urban African American families. *Child Development*.

[45] Schwinn TM, Schinke SP, Trent DN (2010). Substance use among late adolescent urban youths: mental health and gender influences. *Addictive Behaviors*.

[46] Piran N (2004). Prevention series. Teachers: on "being" (rather than "doing") prevention. *Eating Disorders*.

[47] Stice E, Shaw H, Marti CN (2007). A meta-analytic review of eating disorder prevention programs: encouraging findings. *Annual Review of Clinical Psychology*.

